# Maternal lipidomic signatures in relation to spontaneous preterm birth and large-for-gestational age neonates

**DOI:** 10.1038/s41598-021-87472-9

**Published:** 2021-04-14

**Authors:** Max T. Aung, Pahriya Ashrap, Deborah J. Watkins, Bhramar Mukherjee, Zaira Rosario, Carmen M. Vélez-Vega, Akram N. Alshawabkeh, José F. Cordero, John D. Meeker

**Affiliations:** 1 Department of Biostatistics, University of Michigan, School of Public Health, Ann Arbor, MI USA; 2 Department of Environmental Health Sciences, University of Michigan, School of Public Health, 1415 Washington Heights, Ann Arbor, MI 48109 USA; 3 Department of Epidemiology, University of Michigan, School of Public Health, Ann Arbor, MI USA; 4 University of Puerto Rico Graduate School of Public Health, UPR Medical Sciences Campus, San Juan, Puerto Rico, USA; 5 College of Engineering, Northeastern University, Boston, MA USA; 6 Department of Epidemiology and Biostatistics, University of Georgia, Athens, GA USA

**Keywords:** Biomarkers, Risk factors, Epidemiology

## Abstract

Lipidome-wide metabolites may be useful biomarkers of pregnancy outcomes. We sought to characterize maternal lipidomic signatures associated with preterm birth and neonatal anthropometric parameters. Plasma samples were collected 24–28 weeks gestation, and lipidomic profiling was quantified using high-performance liquid chromatography tandem mass spectrometry. Lipid metabolites were analyzed individually and as whole lipid classes and subgroups based on degree of hydrocarbon chain saturation. Associations were estimated using linear and logistic regression. After false discovery adjustment (q < 0.15), four plasmenyl-phosphatidylethanolamines and three free fatty acids associated with increased risk for spontaneous preterm birth. Five phosphatidylinositols, two phosphatidylglycerols, and one phosphatidic acid were associated with large for gestational age neonates. The saturated plasmenyl-phosphatidylethanolamines held the association with increased risk for spontaneous preterm birth. Both the mono- and poly-unsaturated free fatty acids held the association for increased risk for spontaneous preterm birth. Mono- and poly-unsaturated phosphatidylinositols were associated with large for gestational age neonates. Whole lipid classes (plasmenyl-phophatidylcholines and plasmenyl-phosphatidylethanolamines) were associated with increased risk for large for gestational age at delivery. This study provides evidence that finer omics-scale analysis of the maternal lipidome may be more informative biomarkers of pregnancy outcomes compared to whole class level lipid analysis.

## Introduction

Adequate regulation of maternal lipid metabolism is essential for the progression of a healthy pregnancy and fetal growth. Lipids are generally defined as insoluble organic compounds, and their unique chemical structure is used to classify individual lipid compounds into organizational groups. At the broadest level, some lipid classes of biological interest include phospholipids, glycerides, sterols, and sphingolipids. The structural differences across lipid classes partly define unique biological functions. For example, polymerized glycerides such as triglyceride are stored in adipocytes and released through hormone signaling for energy production^1^. Phospholipids on the other hand have polar heads covalently bound to non-polar hydrocarbon tails, making them a core structural component for lipid membranes and associated integral peptides^2^. Within the sterol class of lipids, cholesterol contributes towards membrane structure maintenance and signal transduction^3^. Finally, sphingolipids are unique in that they contain an amino group bound to hydrocarbon chains, and some are involved in regulating inflammation and immune responses^4^. In addition to these differences across lipid classes, there are also functional differences within the same lipid classes. For example, unique polar head groups differentiate phospholipids. Phospholipids with inositol head groups contribute towards key cellular processes such as migration, proliferation, senescence, and apoptosis^3^. Whereas phosphatidylserines in brain tissue are critical for facilitating neuronal signaling pathways and neurotransmitter release^5^. The diversity of lipids and their functions underscore the importance of investigating them in detail to better understand their signatures in pregnancy.

Maternal lipid profiles change dynamically across the duration of gestation. For example, maternal adipose tissue is increasingly active in the anabolism of circulating lipids during the first and second trimesters, resulting in accumulation and storage of fatty acids, triglycerides, phospholipids, and cholesterols^6,7^. Conversely, during the third trimester, there is an upregulation of lipolysis, resulting in catabolism and decomposition of lipid stores^6,7^. In both processes—accumulation and decomposition of lipid stores—there is a complex matrix of circulating free and vesicular lipids that facilitate crosstalk between the maternal immune-endocrine systems and placenta^8^. Lipidomic analysis affords greater insight into the vast repertoire of lipid metabolites and has potential utility towards defining biomarker signatures of disease states such as inflammation or metabolic dysfunction. Furthermore, individual lipid metabolites can be leveraged to construct broader levels of organizational hierarchies, including whole lipid classes and subgroups of lipid classes based on the degree of saturation in hydrocarbon chains.

Estimation of lipidomic biomarker signatures can have predictive utility for a wide range of pregnancy outcomes. For example, preterm birth—delivery prior to 37 weeks gestation—remains a persistent public health concern and affects approximately 10% of live births^9^. Furthermore, social factors such as environmental injustice, income inequality, and systemic racism have led to disparities in rates of adverse pregnancy outcomes^10^. In Puerto Rico, the rate of preterm birth in 2019 was 11.9%^11^. It is critical for emerging omics-scale studies to include underrepresented communities that are historically neglected in order to better understand and prevent health disparities. Consequently, characterization of lipidomic signatures among preterm birth cases in Puerto Rico can provide insight on early markers of disease and inform behavioral and therapeutic interventions. Additionally, maternal lipid profiles are responsive to extracellular vesicles and signaling molecules secreted from the placenta^8^. Therefore, lipidomic biomarker signatures can also yield utility in estimation of fetal growth and neonatal anthropometric parameters.

The primary objective of this study was to estimate lipidome-wide associations for multiple pregnancy outcomes, including preterm birth, spontaneous preterm birth, gestational age at delivery, birthweight, and small and large for gestational age neonates. The secondary objective of this study was to construct hierarchies of lipid organization: whole lipid classes and subgroups based on hydrocarbon chain saturation. Our overarching hypothesis is that physiological states underlying adverse pregnancy outcomes would be accompanied by unique lipidomic signatures. For example, systemic inflammation and hormone synthesis may be linked to lipid metabolism, both of which are critical for ensuring adequate progression of a healthy pregnancy. Thus, the motivation to investigate lipid signatures of adverse pregnancy outcomes will have broad applicability towards future studies seeking to characterize mechanisms and early biomarkers of reproductive health outcomes.

## Results

### Descriptive statistics

Table 1 reports demographic and health characteristics of the study sample. The mean age of study participants at the time of enrollment was 26.4 (standard deviation = 5.7). A majority of participants (77%) had some level of higher education experience. In this sample, 44% of participants reported that they were unemployed, and 75% of participant household incomes were below $50 k. Approximately 52% of participants had pre-pregnancy body mass index of greater than 25 kg/m^2^. Most participants did not report smoking (98%) or drinking (92%) during pregnancy.

**Table 1:** Demographic characteristics of study participants recruited between 24 and 28 weeks gestation from the Puerto Rico Testsite for Exploring Contamination Threats (PROTECT) cohort (n = 100).

Variable	Mean (SD)
Maternal age at enrollment (years)	26.4 (5.7)
Gravidity (# pregnancies)	2 (1)
**Insurance type**	**N (%)**
Private	58 (58%)
Public (Mi Salud)	38 (38%)
Missing	4 (4%)
**Maternal education (years)**	
High school/GED	23 (23%)
Some College or technical school	32 (32%)
College degree	33 (33%)
Masters degree or higher	12 (12%)
Missing	-
**Income Status (US $)**	
< $10,000	31 (31%)
≥ $10,000 to < $30,000	24 (24%)
≥ $30,000 to < $50,000	20 (20%)
≥ $50,000	10 (10%)
Missing	15 (15%)
**Marital status**	
Single	23 (23%)
Married or living together	77 (77%)
Missing	-
**Gravidity (# pregnancies)**	
0	48 (48%)
1	33 (33%)
> 1	19 (19%)
Missing	–
**Pre-pregnancy BMI (kg/m**^**2**^**)**	
≤ 25	48 (48%)
> 25 to ≤ 30	33 (33%)
> 30	19 (19%)
Missing	–
**Employment status**	
Employed	55 (55%)
Unemployed	44 (44%)
Missing	1 (1%)
**Smoking**	
Never	86 (86%)
Ever	12 (12%)
Current	2 (2%)
Missing	–
**Exposure to second hand smoking**	
None	80 (80%)
Up to 1 h	5 (5%)
More than 1 h	11 (11%)
Missing	4 (4%)
**Alcohol consumption**	
None	51 (51%)
Before pregnancy	41 (41%)
Yes within the last few months	6 (6%)
Missing	2 (2%)
**Infant sex**	
Female	49 (49%)
Male	51 (51%)
Missing	–

Supplementary Table 1 summarizes the lipid species classes, structures, and the number of lipids annotated from lipidomic analysis. The most abundant lipid classes in this analysis included (1) triacylglycerol, formed by linking fatty acids with an ester linkage to three alcohol groups in glycerol, (2) phosphatidylcholine, a class of phospholipid that are composed of a choline head group and glycerophosphoric acid, and (3) sphingomyelin, which consists of a phosphocholine head group, a sphingosine, and a fatty acid (Supplementary Table 1).

**Figure 1:**
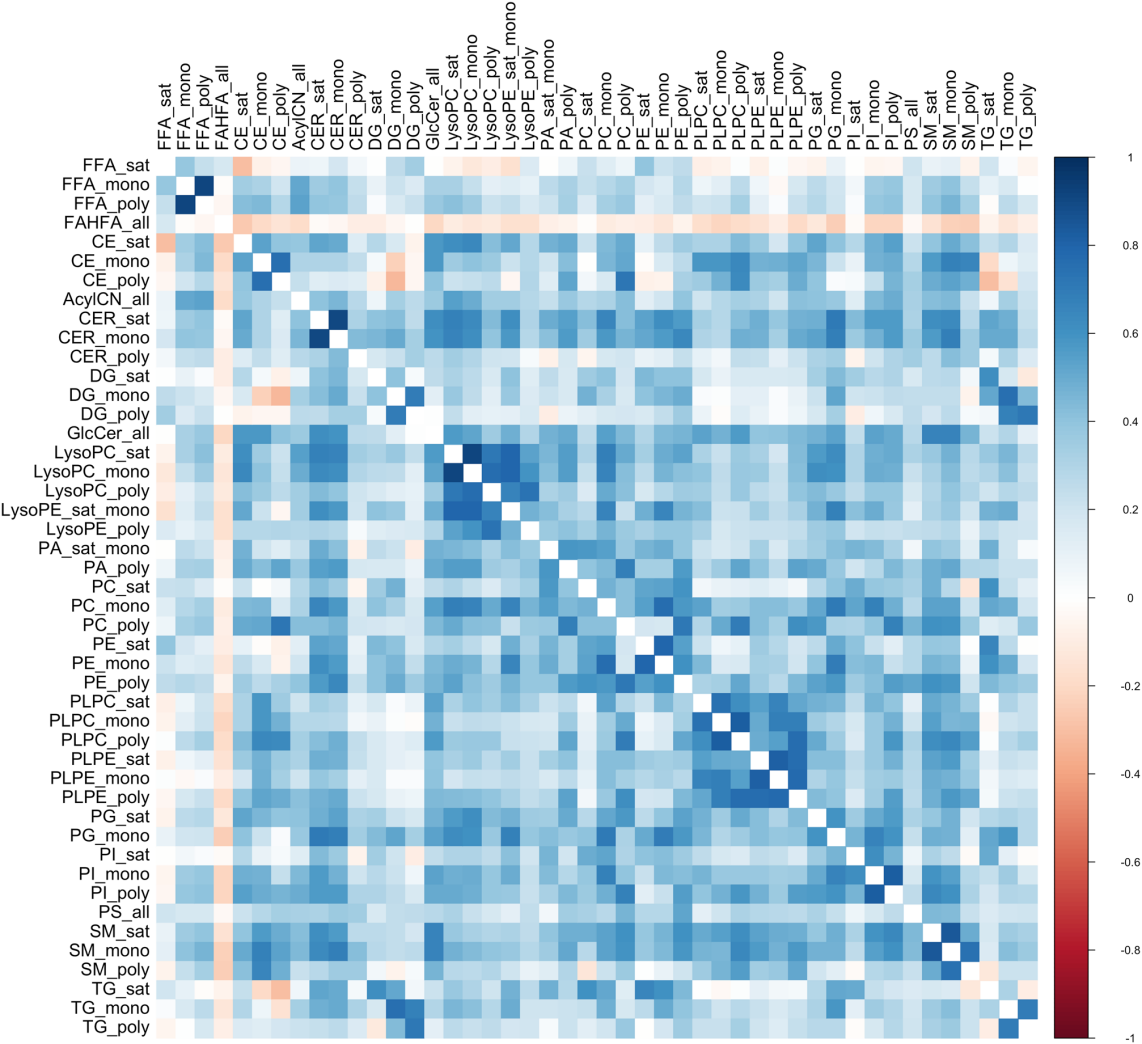
Correlation matrix among lipid hydrocarbon saturation subgroups.

Spearman’s ρ ranged from − 0.74 to 0.97 among all lipid molecules (Supplemental Table 2). The greatest negative correlations were observed between diglycerides and plasmenyl-phosphatidylcholines, while the greatest positive correlations were observed amongst the triglycerides (Supplemental Table 2). When lipids were organized and summed based on their saturation subgroups, we observed Spearman’s ρ ranging from − 0.33 to 0.92 (Fig. 1). The greatest negative correlation was observed between mono-unsaturated diglycerides and poly-unsaturated ceramides, while the greatest positive correlation was observed between mono-unsaturated and saturated lyso-phosphatidylcholines (Fig. 1).

### Lipidome-wide metabolite signatures of pregnancy phenotypes

**Figure 2:**
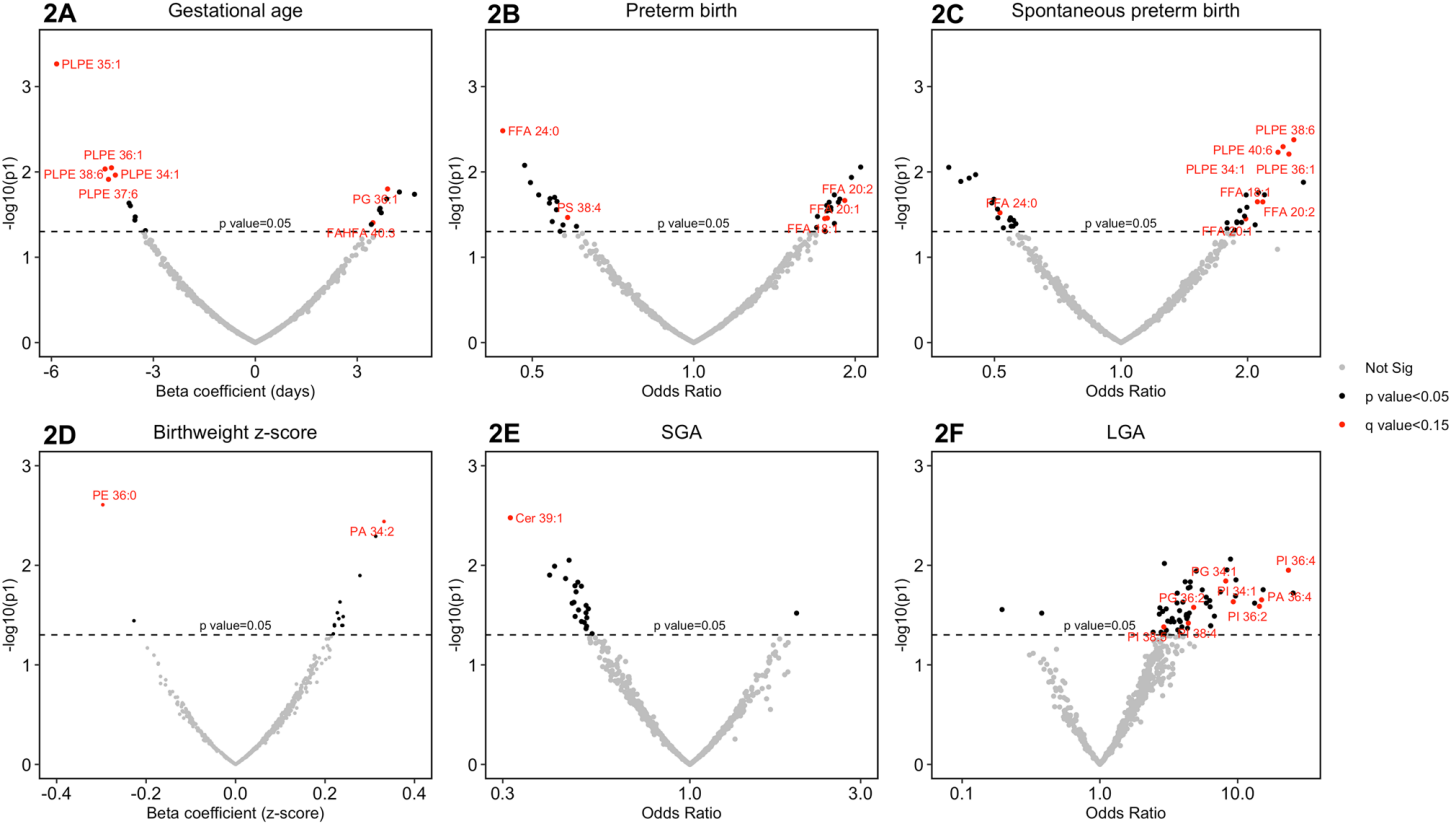
Volcano plots of associations [-log_10_(p-values)] between lipidome-wide metabolites and pregnancy outcomes: (**A**) gestational age at delivery; (**B**) preterm birth (n_cases_ = 31,n_controls_ = 69); (**C**) spontaneous preterm birth (n_cases_ = 19,n_controls_ = 69); (**D**) birthweight z-score; (**E**) small for gestational age [SGA] (n_cases_ = 16,n_controls_ = 75); (**F**) large for gestational age [LGA] (n_cases_ = 9,n_controls_ = 75). Point estimates labeled in red are those that were adjusted for false discovery rate (q-value < 0.15). Models adjusting for maternal age, maternal education, fetal sex, pre-pregnancy BMI, and weight gain.

Figure 2A–F are volcano plots indicating the magnitude of effect estimates and -log_10_(p-value) for each lipid in association with pregnancy phenotypes. All annotated estimates in Fig. 2A–F that are highlighted in red font are those that existed after adjusting for false discovery rate (q-value < 0.15). There were five lipid metabolites from the plasmenyl-phosphatidylethanolamine class associated with reduced gestational age at delivery (Fig. 2A). One phosphatidylglycerol and one hydroxy fatty acid derivative were associated with increased gestational age at delivery (Fig. 2A). Focusing on overall preterm birth, we observed that three free fatty acid lipids were associated with increased risk (Fig. 2B). Two lipid metabolites—one from the free fatty acid class and the other from the phophatidylserine class—were associated with reduced risk for overall preterm birth (Fig. 2B). Disaggregation of spontaneous preterm birth revealed four plasmenyl-phosphatidylethanolamines and three free fatty acids associated with increased risk (Fig. 2C). There was one free fatty acid that was associated with lower risk of spontaneous preterm birth (Fig. 2C).

For the continuous outcome birthweight z-score, one lipid metabolite from the phosphatidic acid class was associated with higher birthweight z-score while one metabolite from the phosphatidylethanolamine class was associated with lower birthweight z-score (Fig. 2D). Contextualizing neonatal size by gestational age revealed one ceramide lipid that was associated with decreased risk of being small for gestational age at delivery (Fig. 2E). Conversely, five lipids from the phosphatidylinositol class, two from the phosphatidylglycerol class, and one from the phosphatidic acid class were associated with increased risk for being large for gestational age at delivery (Fig. 2F). Across all of the phenotypes that we focused on, we observed very little overlap in associated lipid metabolites between preterm birth and the fetal anthropometric outcomes.

### Saturation subgroup associations with pregnancy phenotypes

**Figure 3:**
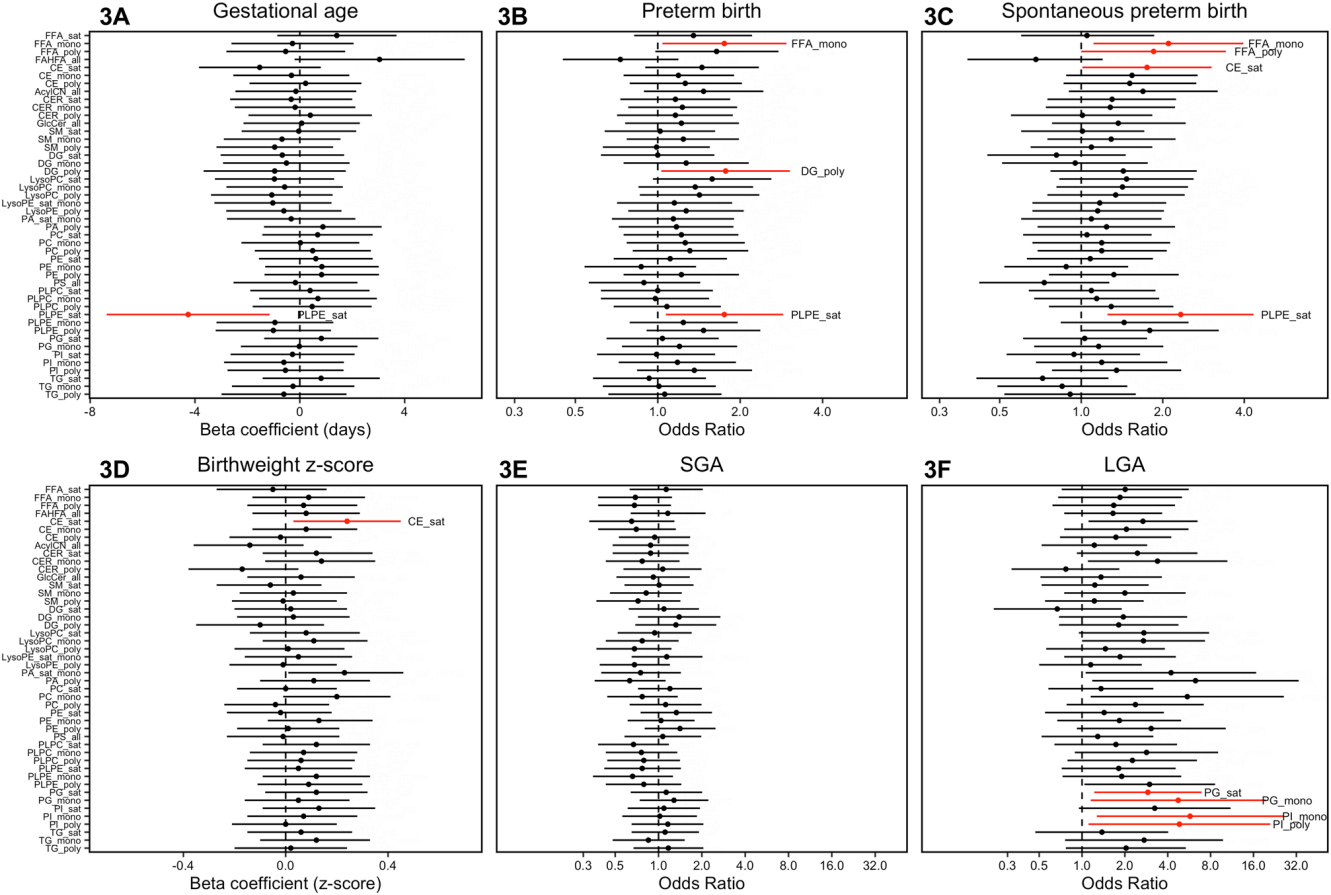
Forest plots of effect estimates of associations between hydrocarbon chain subgroups of lipid classes and pregnancy outcomes: (**A**) gestational age at delivery; (**B**) preterm birth (n_cases_ = 31,n_controls_ = 69); (**C**) spontaneous preterm birth (n_cases_ = 19,n_controls_ = 69); (**D**) birthweight z-score; (**E**) small for gestational age [SGA] (n_cases_ = 16,n_controls_ = 75); (**F**) large for gestational age [LGA] (n_cases_ = 9,n_controls_ = 75). Models adjusting for maternal age, maternal education, fetal sex, pre-pregnancy BMI, and weight gain. Associations highlighted in red have p-values < 0.05, but all associations had corresponding q-values exceeding 0.2.

Figures 3A–F are forest plots of associations with pregnancy phenotypes upon summation of individual lipid metabolites based on the degree of saturation in hydrocarbon tails. For gestational age at delivery, we observe only saturated plasmenyl-phophatidylethanolamines were associated with decreased gestational age at delivery (Fig. 3A). Three different lipid subgroups (mono-unsaturated free fatty acids, poly-unsaturated diglycerides, and saturated plasmenyl-phophatidylethanolamines) were associated with increased risk for overall preterm birth (Fig. 3B). Focusing on spontaneous preterm birth, we observed that mono- and poly-unsaturated free fatty acids, saturated ceramides, and saturated plasmenyl-phosphatidylethanolamines were all associated with increased risk (Fig. 3C). Saturated ceramides were associated with higher birthweight z-score (Fig. 3D). At this level of lipid organization, we did not observe any notable associations with small-for-gestational age at delivery (Fig. 3E). However, saturated and mono-unsaturated phosphatidylglycerols, and mono- and poly-unsaturated phosphatidylinositols were associated with increased risk for large-for-gestational age at delivery (Fig. 3F).

### Whole lipid classes associations with pregnancy phenotypes

**Figure 4:**
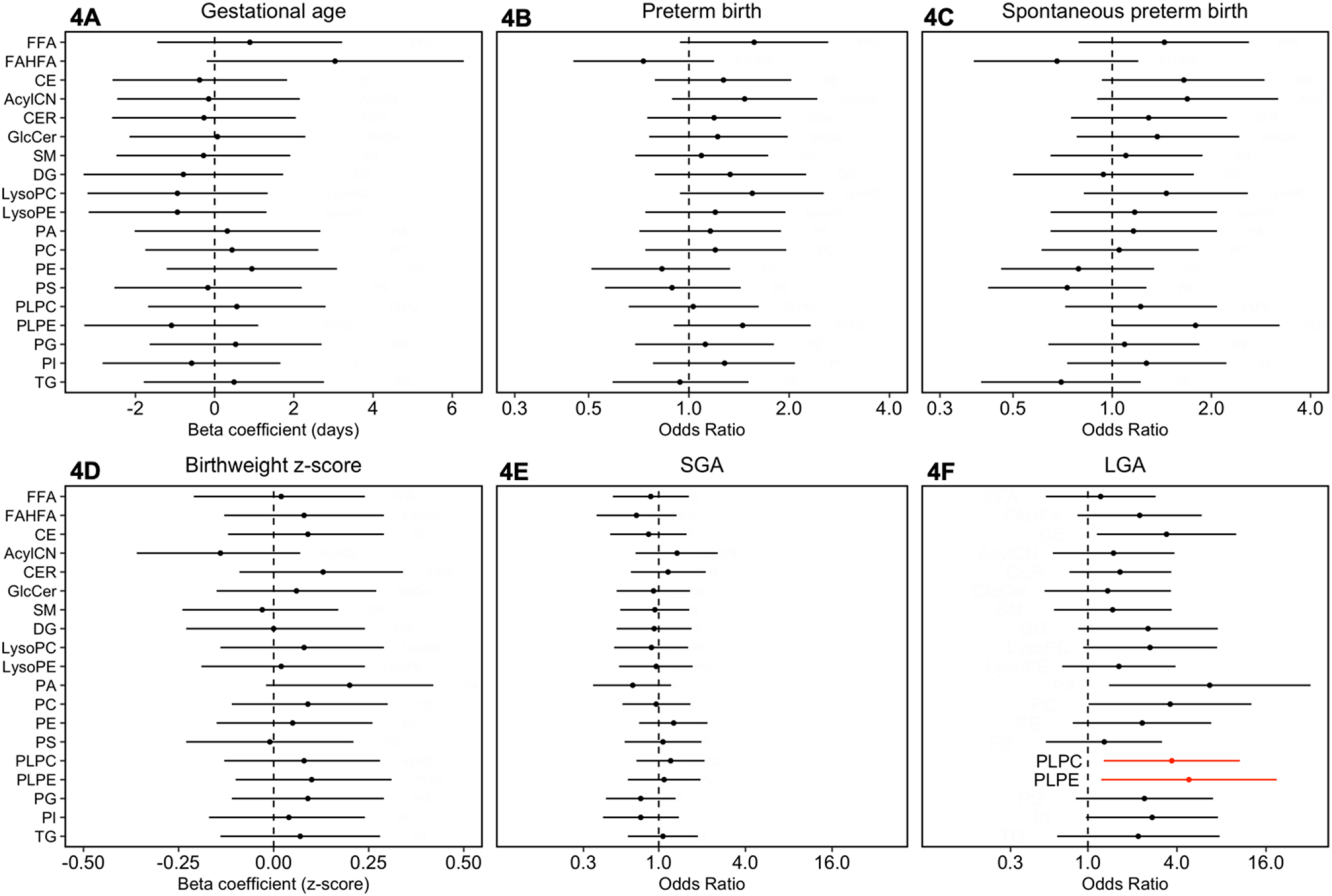
Forest plot of effect estimates of associations between whole lipid classes and pregnancy outcomes: (**A**) gestational age at delivery; (**B**) preterm birth (n_cases_ = 31,n_controls_ = 69); (**C**) spontaneous preterm birth (n_cases_ = 19,n_controls_ = 69); (**D**) birthweight z-score; (**E**) small for gestational age [SGA] (n_cases_ = 16,n_controls_ = 75); (**F**) large for gestational age [LGA] (n_cases_ = 9,n_controls_ = 75). Models adjusting for maternal age, maternal education, fetal sex, pre-pregnancy BMI, and weight gain. Associations highlighted in red have p-values < 0.05, but all associations had corresponding q-values exceeding 0.2.

Figure 4A–F are forests plots of associations between whole lipid classes and pregnancy phenotypes. In this analysis, we observe largely null or suggestive associations when clustering all lipid metabolites into their highest level of organization. There were two lipid classes—plasmenyl-posphatidylcholines and plasmenyl-phosphatidylethanolamines—that were associated with increased risk for large-for-gestational age at delivery (Fig. 4F).

### Lipid class selection in multi-lipid models

Sparse-group lasso showed that the most important and predictive lipid class for overall preterm birth was free fatty acids (Supplemental Fig. 1). For spontaneous preterm birth, sparse-group lasso selected both free fatty acids and plasmenyl-phophatidylcholines as the most important lipid classes (Supplemental Fig. 2). For large for gestational age neonates, sparse-group lasso identified free fatty acids and phosphatidylinositols as most predictive (Supplemental Fig. 3).

## Discussion

In this extensive lipidomic analysis, we investigated three hierarchical levels of lipid organization: (1) individual lipidome-wide metabolites, (2) subgroup clustering based on hydrocarbon chain saturation, and (3) whole lipid classes. Using these organizational hierarchies of lipids, we sought to explore their signatures in association with multiple pregnancy phenotypes: preterm birth and its subtype spontaneous preterm birth, gestational age at delivery, birthweight, and neonatal size relative to gestational age at delivery. We found that at the broadest level of whole lipid classes, there is not substantive evidence of associations in this sample. Upon evaluation of lipid saturation subgroups, we observed more pronounced relationships with multiple phenotypes. At the lipidome-wide level of individual metabolites, we observed multiple single metabolites associated with each pregnancy phenotype. Phospholipid classes appeared to have the greatest number of metabolite specific signatures with multiple pregnancy outcomes. The notable theme in our findings was the variation in signatures among distinct phospholipid classes (phosphatidic acids, plasmenyl-phosphatidylethanolamines, phosphatidylcholines, and phosphatidylglycerols). These findings support the need to measure lipid metabolites at a finer omics-scale through lipidome-wide measurement to identify important lipid-derived biomarkers of adverse pregnancy outcomes not captured with the cruder yet more common class-level measurements.

The dominant lipid signature for overall preterm birth was from lipid metabolites belonging to the free fatty acid class. This class of lipids was also selected by sparse-group lasso, further underscoring its importance relative to other lipid classes. Free fatty acids include several essential compounds such as arachidonic acid, a major precursor for immuno-potent metabolites such as eicosanoids^12^. The increased risk for preterm birth among a lipidome-wide quantification of free fatty acids is circumstantially consistent with previous findings where we observed increased risk for preterm birth in association with eicosanoids^13^. Other studies have also observed that free fatty acids measured at a general non-specific level were associated with preterm delivery^12,14,15^. When spontaneous preterm birth was disaggregated from the overall preterm birth phenotype, associations involving free fatty acids remained, but the signature of plasmenyl phospholipids was substantially stronger and more significant. Additionally, sparse-group lasso also selected this lipid class as an important predictor of spontaneous preterm birth. This suggests that the metabolism and circulation of plasmenyl phospholipids may be more closely linked to spontaneous labor or premature rupture of the membranes. Overall, these findings provide further evidence that biomarker signatures are differentiated when evaluating general preterm birth compared to more precise etiologically linked phenotypic subclasses.

In a lipidomic profiling study of spontaneous preterm birth (n_cases_ = 16; n_controls_ = 32) based in Ireland, Morillon et al. (2020) observed that several metabolites (measured at 20 weeks’ gestation) within the phosphatidylcholine and phosphatidylethanolamine classes were significantly lower among cases. This study also found that multiple sphingolipids were downregulated in cases of spontaneous preterm birth^16^. Another lipidomic study preterm birth focusing exclusively on Black women (n_cases_ = 9; n_controls_ = 20) in the U.S. Midwest observed that lipid metabolites (measured 9–25 weeks’ gestation) in the phosphatidylcholine class were downregulated in preterm birth cases while triglycerides were upregulated^17^. Potential factors influencing differences in exact lipid signatures could be due to demographic differences between our study and the comparative studies. Furthermore, the timing of lipidome measurement was different as well. We focused on samples collected in the second and third trimesters, while the comparison studies measured the maternal lipidome earlier in pregnancy, during the first and second trimesters.

Although we did not observe consistency with previous studies for findings of sphingolipids or phosphatidylcholines, it is interesting that we observed several signatures with plasmenyl-phosphatidylethanolamines, which are structurally similar to phosphatidylethanolamines. Both of these lipid classes are glycerophospholipids that provide structural integrity to lipid bilayers and are necessary for proper functionality of integral membrane proteins^18^. However, plasmenyl-phosphatidylethanolamines are distinct due to the alkyl ether functional group. These lipids are found in the largest proportions within cells of brain tissues, and mechanistic studies have found that deficiencies in plasmenyl-phosphatidylethanolamines are associated with neurodegenerative disorders, cardiovascular disease, and respiratory conditions, potentially through chronic inflammatory states^18^. The direction of association in our study indicated that higher concentrations—rather than deficiency—was associated with increased risk for overall and spontaneous preterm birth. This finding may suggest that elevation in circulating plasmenyl-phosphatidylethanolamines may be a consequence of tissue damage or inflammatory states preceding spontaneous preterm birth. Additional longitudinal studies are needed to evaluate this hypothesis.

There was very little overlap in maternal lipid metabolites when comparing neonatal anthropometric outcomes (small-for-gestational age, large-for-gestational age, and birthweight) and non-anthropometric outcomes (overall preterm birth, spontaneous preterm birth, and gestational age at delivery). This finding is interesting because it gives initial evidence that neonatal anthropometric parameters may be more sensitive to different maternal lipid profiles compared to preterm birth. The significance of this finding is that potential therapeutic and dietary interventions may need to consider different lipid metabolite biomarkers when evaluating risk factors for neonatal versus maternal pregnancy phenotypes.

In small for gestational age neonates, we only observed lower levels of one maternal ceramide metabolite. This finding parallels the lack of lipids associated with decreased birthweight z-score. Perhaps the timing of lipidomic measurement in this study is not sensitive at displaying lipid signatures for these particular neonatal outcomes. Additionally, the sample size of cases that were small for gestational age was low (n = 16), limiting power to detect notable lipid signatures. One previous study based in Spain measured maternal lipids at the time of delivery, and detected lower concentrations of circulating cholesterols, triglycerides, and phosphatidylcholines in women with small for gestational age neonates (n_cases_ = 52, n_controls_ = 28)^19^. Unlike other comparison studies cited earlier, this study quantified lipids using nuclear magnetic resonance, which differed from our quantitative approach and could also explain differences in observed associations^19^. Another study based in the United Kingdom measured maternal lipid metabolites at 15 weeks gestation and observed higher concentrations from the lysophosphatidylcholine and sphingolipid classes among small for gestational age neonates (n_cases_ = 40, n_controls_ = 40)^20^. The inconsistencies in findings for small for gestational age neonates across existing studies and our current study may also be influenced by timing of lipidomic measurements and differences in geographic and social factors.

Although the sample size of large for gestational age neonates was small (n = 9), among the neonatal outcomes, we observed the greatest lipid signature when evaluating this outcome—multiple phosphatidylinositols and one phosphatidylglycerol were associated with increased risk. Giving birth to large for gestational age neonates is an important phenotype to focus on, given that these pregnancies are associated with co-morbidities such as maternal obesity, gestational diabetes, and gestational weight gain^21,22^. Maternal phosphatidylinositol and phosphatidylglycerol signatures may be important signals linking these interconnected phenotypes. From our knowledge, there are no existing lipidome-wide analyses of large for gestational age neonates. Nonetheless, phosphatidylinositols are present in placental exosomes, which are extracellular vesicles that may play a critical role in placental development and promoting maternal immunotolerance during pregnancy^23^. Therefore, physiological states such as systemic inflammation or hormone disruption can potentially result in perturbations in exosomal phosphatidylinositol signatures in the maternal circulation^7^. This hypothesis can be further tested with intentional measurement of not only maternal lipidome signatures, but also isolated exosome fractions from the maternal circulation.

The major limitation in our study design is the modest sample size. However, a majority of the previously conducted lipidome-wide association studies cited were focused on smaller sample sizes. This exploratory analysis builds on the methodological foundation of previous studies and provides evidence for scaling up future larger replication studies. Another limitation in our study is that there are unmeasured confounders that may affect the estimation of associations in our study. For example, our study did not evaluate maternal dietary patterns, which could be driving both lipidomic signatures, pregnancy outcomes, and fetal growth. We partially accounted for this unmeasured confound by adjusting for maternal pre-pregnancy BMI and weight gain during pregnancy. Additionally, adjustment for diet may not be appropriate in this setting, given that internal lipid profiles are directly dependent on dietary intake, in which case mediation analysis may be a more appropriate method to account for the relationship between diet, lipidomic profiles, and pregnancy outcomes. Furthermore, adjustment for weight gain during pregnancy provides a more precise measurement of physiological changes due to diet, rather than dietary food intake questionnaires. Another limitation in our study is that the lipidomic biomarkers we measured were restricted to maternal circulation, therefore we are unable to evaluate tissue specific lipid profiles. Future studies should evaluate other tissues such as placenta and amniotic fluid, to evaluate the degree of consistency or divergence in lipid signatures for pregnancy phenotypes. Our study was also limited by having only one time point for measurement of the maternal lipidome, therefore we were unable to evaluate windows of vulnerability during pregnancy. Given that lipid profiles change across the duration of pregnancy, certain periods may be more insightful for the etiology of preterm birth and neonatal anthropometric outcomes.

Our study contained several strengths that should be underlined. First, a unique attribute about our study is the demographic representation of our study sample. We focused on an underrepresented U.S. community which not only diversifies modern omics-scale study populations, but also critically informs data driven policy development to reduce health disparities within the U.S. Another strength of our study was the exhaustive repertoire of metabolites that were measured within the maternal lipidome, which has the potential to yield important biomarkers of adverse pregnancy outcomes. In the lipidomic biomarker space, we also pursued creative approaches to develop hierarchical organization of metabolites based on whole lipid classes and hydrocarbon saturation subgroups. This approach informs future studies that may be limited in their ability to measure the entire lipidome, and therefore must prioritize specific lipid classes and subgroups.

In conclusion, this study provides evidence that finer omics-scale analysis of the maternal lipidome may be more informative biomarkers of adverse pregnancy outcomes compared to cruder levels of lipid quantification. Lipidomic measurement may advance precision health profiling of maternal and fetal outcomes, which warrants subsequent replication studies. Furthermore, this study discusses major gaps in lipidomic research, chiefly, the need to evaluate repeated measurements of the maternal lipidome and also quantification across multiple tissue types. Future studies that pursue these research gaps will advance scientific discovery in characterizing the etiology of adverse pregnancy outcomes.

## Methods

### Study sample

This study sample is an exploratory subset of the Puerto Rico Testsite for Exploring Contamination Threats (PROTECT) cohort. From the larger cohort of 812 pregnant women, we randomly sampled 100 women for lipidome-wide analysis with the goal of achieving a 1:2 case–control ratio for preterm birth. The PROTECT cohort began recruitment in 2010 through funding from the National Institute of Environmental Health Sciences Superfund Research Program. Study participants were recruited in the first or early second trimester of pregnancy (median 14 weeks gestation). Inclusion criteria for recruitment included: participant age between 18 and 40 years; residence in the Northern Karst aquifer region; disuse of oral contraceptives three months before pregnancy; disuse of in vitro fertilization; and no indication of medical records for major obstetrical complications, including pre-existing diabetes. The research protocol was approved by the Ethics and Research Committees of the University of Puerto Rico and participating clinics, the University of Michigan, Northeastern University, and the University of Georgia. The study was described in detail to all participants, and informed consent was obtained prior to study enrollment. All methods within this study were performed in accordance with the relevant guidelines and regulations approved by the Ethics and Research Committees.

### Pregnancy phenotypes

All of the birth outcome data were extracted from medical records. The American Congress of Gynecologists recommendations for best obstetrical estimate to calculate the gestational age for complete pregnancies^24^ were used in our study as previously described^25,26^. Per common practice, preterm birth was defined as delivery < 37 completed weeks of gestation. Based on the presentation of preterm delivery, preterm birth was further classified as spontaneous preterm birth (presentation of premature rupture of the membranes, spontaneous preterm labor, or both) and non-spontaneous preterm birth (preterm births with preeclampsia or with both artificial membrane rupture and induced labor). We included overall and spontaneous preterm birth as two of the birth outcomes in our analysis.

Birthweight z-scores (defined as the number of standard deviations by which a birthweight is above or below the mean) are commonly used to compare individual birthweights within the cohort^27^. Gestational age- and sex- specific birthweight z-score were constructed according to the INTERGROWTH-21st standards^28^. Small for gestational age (SGA) births were defined as those below the 10th percentile of birthweight z-scores. Large for gestational age (LGA) births were defined as those above the 90th percentile of birthweight z-scores.

### Lipidome measurement

Plasma lipids are extracted from blood samples provided by women between 24 and 28 weeks gestation using a modified Bligh-Dyer method^29^ using liquid–liquid extraction at room temperature after spiking with internal standards. Analysis of lipids was performed on reversed phase high-performance liquid chromatography (HPLC), followed by mass spectrometry (MS) analysis that alternates between MS and data-dependent MS2 scans using dynamic exclusion in both positive and negative polarity and yields excellent separation of all classes of lipids. The lipids are quantified using Multiquant and normalized by internal standards.

For chromatography, a Shimadzu CTO-20A Nexera X2 UHPLC systems equipped with a degasser, binary pump, thermostated autosampler, and a column oven for chromatographic separation were used. A linear gradient using solvents was created over the 20-min total run time. Solvent A contained acetonitrile/water (40:60, v/v) with 10 mM ammonium acetate. Solvent B contained acetonitrile/water/isopropanol (10:5:85, v/v/v) with 10 mM ammonium acetate. For the first ten minutes, a 60% Solvent A and 40% Solvent B was used. For the next seven minutes, the gradient increased in a linear fashion to 100% Solvent B. Thereafter the system was switched back to 60% Solvent B and 40% Solvent A for 3 min. The flow rate used for these experiments was 0.4 mL/min and the injection volume was 5μL. The column was equilibrated for three minutes before the next injection and run at a flow rate of 0.4 uL/min for a total run time of 20 min^30^.

Samples were ionized in positive and negative ionization model using a Triple TOF 5600 (AB Sciex, Concord Canada). The source voltage was set to 5500 V for positive ionization and 4500 V for negative ionization mode, the declustering potential was set to 60 V, and the source temperature to 450 °C for both modes. The instrument performed one TOF MS survey scan (150 ms) and 15 MS/MS scans with a total duty cycle time of 2.4 s. The mass range in both modes was 50–1200 m*/z*. Measurements are semi-quantitative and are based on relative abundance of peak intensities.

Quality controls are prepared by pooling equal volumes of each sample, in addition to a well characterized plasma pool, and are injected at the beginning and end of each analysis and after every 10 sample injections, to provide a measurement of the system’s stability and performance as well as reproducibility of the sample preparation method^31^.

Lipids are identified using the LipidBlast^32^ library (computer-generated tandem mass spectral library of 212,516 spectra covering 119,200 compounds from 26 lipid compound classes), by matching the product ions MS/MS data. The method allowed us to identify and quantify 587 lipids belonging to 19 different lipid classes, including acylcarnitine (AcyICN), ceramides (CER), cholesterol esters (CE), diacylglycerols (DG), glucosylceramides (GlcCer), free fatty acids (FFA), fatty acid esters of hydroxy fatty acids (FAHFA), lysophosphatidylcholine (LysoPC), lysophosphatidylethanolamine (LysoPE), phosphatidic acid (PA), phosphatidylcholine (PC), phosphatidylethanolamine (PE), phosphatidylglycerol (PG), plasmenyl-phosphatidylcholine (PLPC), plasmenyl-phosphatidylethanolamine (PLPE), phosphatidylinositol (PI), phosphatidylserine (PS), sphingomyelin (SM), and triacylglycerol (TG).

### Statistical analyses

Group sum of the individual lipids’ relative abundance that belong to each lipid class were calculated to construct a total measurement for whole lipid classes. As chain length and number of double bonds in the lipid are of biological interest, sub-group (saturated, mono-unsaturated, poly-unsaturated) sums of lipids’ relative abundance were also created. Descriptive statistics for lipids were computed to examine their distributions. Spearman correlation coefficients were calculated between individual lipids, lipid class, and lipid sub-groups.

Multiple statistical strategies were used to assess relationships between lipid profiles and birth outcomes. The relative abundance of individual lipids were log-transformed due to their skewed distribution. Subsequently the log-transformed individual lipids, sub-group sums, and group sums were normalized among individuals (rows) by standard normal variate normalization. Multiple linear and logistic regression models were fitted to evaluate the association between continuous and binary birth outcomes and the lipidome. Models were adjusted for covariates based on a priori assessment and through model fit diagnostic parameters such as adjusted R^2^ and Akaike information criterion. Final covariates included maternal age, maternal education, fetal sex, pre-pregnancy BMI, and pregnancy weight gain. Individual models were performed for each log-transformed and standardized relative abundance of lipid, creating 587 models for each birth outcome. False discovery rate (FDR) adjusted p-values (q-values), a commonly used method of adjusting for multiple comparisons in lipidomics studies, were used to account for multiple comparisons. Sums for each lipid class (p_c_ = 19) and lipid sub-group (p_g_ = 46) (saturated, mono-unsaturated, poly-unsaturated) were then regressed on each birth outcome.

Regression results were transformed and presented as estimated percentage changes for continuous outcomes (gestational age at delivery and birthweight z-score) and odds ratios for dichotomous outcomes (preterm birth, spontaneous preterm birth, SGA, and LGA) with the corresponding 95% confidence intervals and p values per unit change in lipids z score.

Models were also constructed with all lipids simultaneously using sparse-group lasso (*SGL* package, version 1.3). In this setting, lipids were given a group-level identity according to their class. Sparse-group lasso operates as a variable selection method that utilizes tuning parameters to control for groupwise and within group sparsity among lipids to determine which lipid classes are most predictive of individual outcome variables.

## Supplementary Information

Supplementary Information 1.

Supplementary Information 2.

## Data Availability

Data utilized for this analysis can be obtained by reasonable request by contacting both the first author (MTA, Max.Aung@ucsf.edu) and the corresponding author (JDM, meekerj@umich.edu).

## Abbreviations

PROTECT: Puerto Rico Testsite for Exploring Contamination Threats
SGA: Small for gestational age
LGA: Large for gestational age
HPLC: High-performance liquid chromatography
MS: Mass spectrometry
AcyICN: Acylcarnitine
CER: Ceramides
CE: Cholesterol esters
DG: Diacylglycerols
GlcCer: Glucosylceramides
FFA: Free fatty acids
FAHFA: Fatty acid esters of hydroxy fatty acids
LysoPC: Lysophosphatidylcholine
LysoPE: Lysophosphatidylethanolamine
PA: Phosphatidic acid
PC: Phosphatidylcholine
PE: Phosphatidylethanolamine
PG: Phosphatidylglycerol
PLPC: Plasmenyl-phosphatidylcholine
PLPE: Plasmenyl-phosphatidylethanolamine
PI: Phosphatidylinositol
PS: Phosphatidylserine
SM: Sphingomyelin
TG: Triacylglycerol
