# Supplementary material for: Maternal lipidomic signatures in relation to spontaneous preterm birth and large-for-gestational age neonates
Source: Sci Rep. 2021 Apr 14;11:8115. doi: 10.1038/s41598-021-87472-9 (PMC8046995; doi:10.1038/s41598-021-87472-9)

Title: Maternal lipidomic signatures in relation to spontaneous preterm birth and large-for-gestational age neonates

Authors: Max T. Aung[1], Pahriya Ashrap[2], Deborah J. Watkins[2], Bhramar Mukherjee[1,3], Zaira Rosario[4], Carmen M. Vélez-Vega[4], Akram N. Alshawabkeh[5], José F. Cordero[6], John D. Meeker[2*]

Affiliations:

[1]Department of Biostatistics, University of Michigan, School of Public Health, Ann Arbor, MI
[2]Department of Environmental Health Sciences, University of Michigan, School of Public Health, Ann Arbor, MI
[3]Department of Epidemiology, University of Michigan, School of Public Health, Ann Arbor, MI
[4]University of Puerto Rico Graduate School of Public Health, UPR Medical Sciences Campus, San Juan, Puerto Rico
[5]College of Engineering, Northeastern University, Boston, MA
[6]Department of Epidemiology and Biostatistics, University of Georgia, Athens, GA

Supplementary Table 1 and Figures

Supplementary Table 1. Summary of different lipid classes, structures, and number of lipids annotated from lipidomics analysis

Lipid Class	Abbreviation	Structure	Number
Acylcarnitine	AcylCN	Acyl esters of the quaternary ammonium compound carnitine, containing a carboxyl group and a quaternary ammonium group in the molecule	7
Ceramide	CER	A family of waxy lipid molecules, composed of sphingosine and a fatty acid.	38
Cholesteryl Ester	CE	Ester of cholesterol and the ester bond is formed between the carboxylate group of a fatty acid and the hydroxyl group of cholesterol.	14
Diacylglycerol	DG	Glyceride consisting of two fatty acid chains covalently bonded to a glycerol molecule through ester linkages.	36
Free Fatty Acid	FFA	Consists of a straight chain of an even number of carbon atoms, with hydrogen atoms along the length of the chain and at one end of the chain and a carboxyl group (—COOH) at the other end.	16
Fatty Acid Esters of Hydroxy Fatty Acids	FAHFA	A structurally novel class of endogenous lipids, consists of hydroxy fatty acids (HFAs) acylated by fatty acids	2
Glucosylceramide	GlcCer	A group of glycosphingolipids that consist of a ceramide with a single sugar residue at the 1-hydroxyl moiety	10
Lysophosphatidylcholine	LysoPC	A class of chemical compounds which are derived from partial hydrolysis of phosphatidylcholines, which removes one of the fatty acid groups	23
Lysophosphatidylethanolamine	LysoPE	A class of chemical compounds which are derived from partial hydrolysis of phosphatidylethanolamine, which removes one of the fatty acid groups. They are composed of an ethanolamine head group and glycerophosphoric acid with a various fatty acid located sn-1 position.	9
Phosphatidic Acid	PA	Anionic phospholipids that consist of a glycerol backbone, with, in general, a saturated fatty acid bonded to carbon-1, an unsaturated fatty acid bonded to carbon-2, and a phosphate group bonded to carbon-3.	6
Phosphatidylcholine	PC	A class of phospholipid that are composed of a choline head group and glycerophosphoric acid, with a variety of fatty acids	87
Phosphatidylethanolamine	PE	A class of phospholipid that consists of a combination of glycerol esterified with two fatty acids and phosphoric acid	45
Plasmenyl-Phosphatidylcholine	PLPC	A subclass of ether phospholipids that contain a vinyl ether substituent at the sn-1	55

		position of the glycerol backbone of Phosphatidylcholine	
Plasmenyl-Phosphatidylethanolamine	PLPE	A subclass of ether phospholipids that contain a vinyl ether substituent at the sn-1 position of the glycerol backbone of Phosphatidylethanolamine	43
Phosphatidylglycerol	PG	A glycerophospholipid consists of a L-glycerol 3-phosphate backbone ester-bonded to either saturated or unsaturated fatty acids on carbons 1 and 2	7
Phosphatidylinositol	PI	A class of the phosphatidylglycerides consists of a phosphatidic acid backbone, linked via the phosphate group to inositol (hexahydroxycyclohexane).	13
Phosphatidylserine	PS	A glycerophospholipid which consists of two fatty acids attached in ester linkage to the first and second carbon of glycerol and serine attached through a phosphodiester linkage to the third carbon of the glycerol.	2
Sphingomyelin	SM	Consists of a phosphocholine head group, a sphingosine, and a fatty acid	68
Triacylglycerol	TG	Formed by linking fatty acids with an ester linkage to three alcohol groups in glycerol	106

Supplemental Figure 1. Sparse-group lasso on whole lipid classes for overall preterm birth. The dashed verticle lines indicate the value of log(λ) corresponding to the first time the whole lipid class was excluded from the model as log(λ) increases. The numbers 1 through 20 indicate the following: (1) covariates; (2) free fatty acids; (3) fatty acid esters of hydroxy fatty acids; (4) cholesterol esters; (5) acylcarnitines; (6) ceramides; (7) glucosylceramides; (8) sphingomyelins; (9) diglycerides; (10) lysophosphatidylcholines; (11) lysophosphatidylethanolamines; (12) phosphatidic acids; (13) phosphatidylcholines; (14) phosphatidylethanolamines; (15) phosphatidylserines; (16) plasmenyl-phosphatidylcholines; (17) plasmenyl-phosphatidylethanolamines; (18) phosphatidylglycerols; (19) phosphatidylinositols; (20) triglycerides.

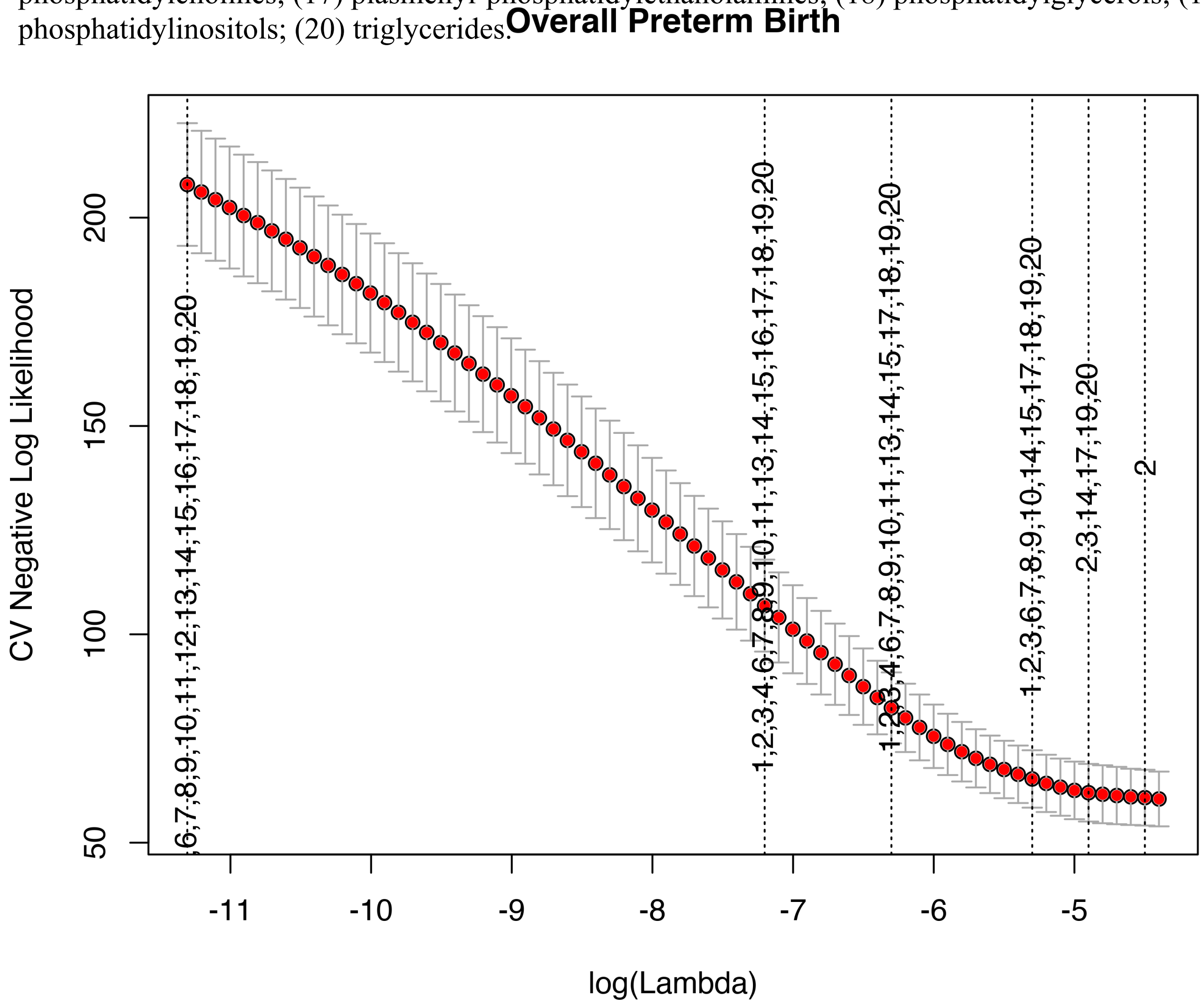

Supplemental Figure 2. Sparse-group lasso on whole lipid classes for spontaneous preterm birth. The dashed verticle lines indicate the value of log(λ) corresponding to the first time the whole lipid class was excluded from the model as log(λ) increases. The numbers 1 through 20 indicate the following: (1) covariates; (2) free fatty acids; (3) fatty acid esters of hydroxy fatty acids; (4) cholesterol esters; (5) acylcarnitines; (6) ceramides; (7) glucosylceramides; (8) sphingomyelins; (9) diglycerides; (10) lysophosphatidylcholines; (11) lysophosphatidylethanolamines; (12) phosphatidic acids; (13) phosphatidylcholines; (14) phosphatidylethanolamines; (15) phosphatidylserines; (16) plasmenyl-phosphatidylcholines; (17) plasmenyl-phosphatidylethanolamines; (18) phosphatidylglycerols; (19) phosphatidylinositols; (20) triglycerides.

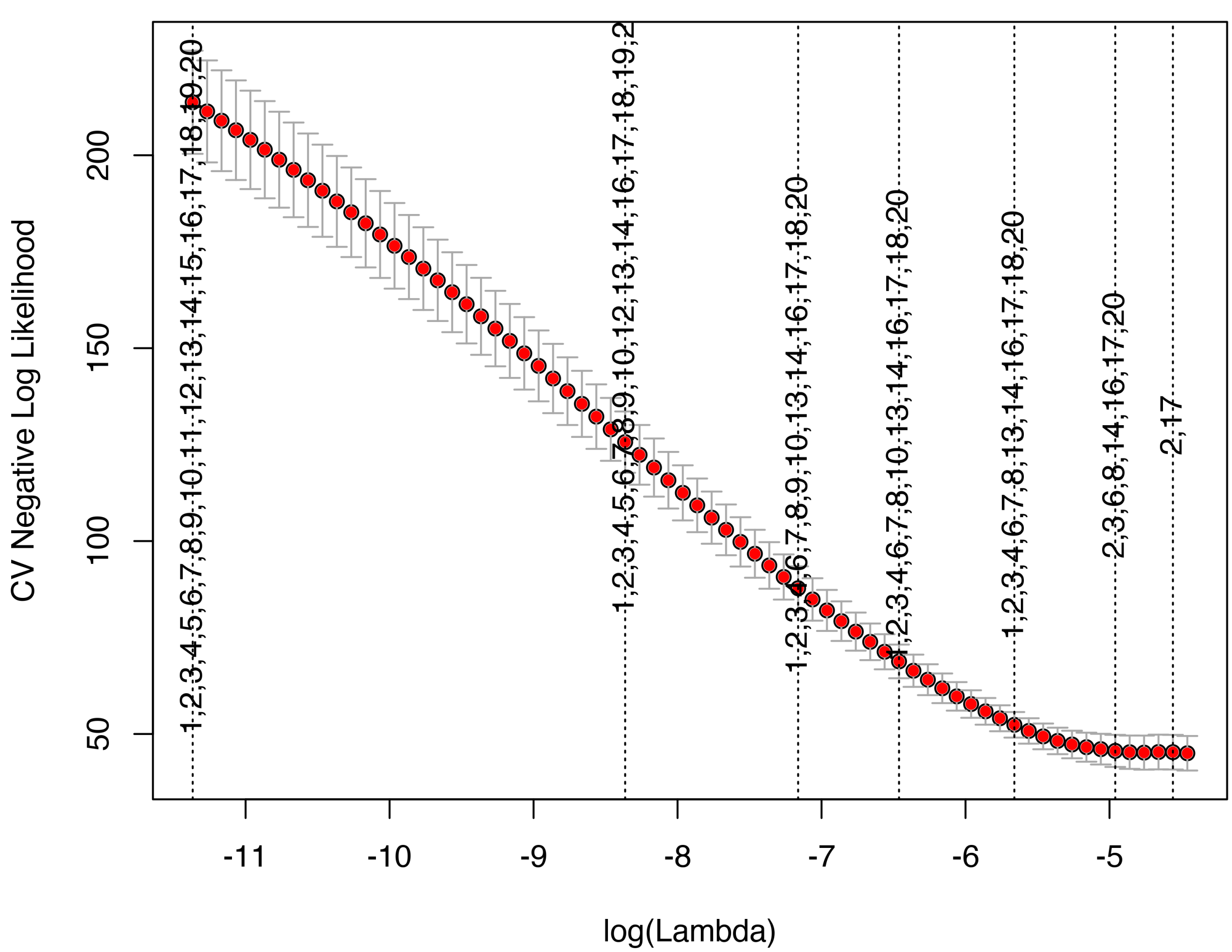

Supplemental Figure 3. Sparse-group lasso on whole lipid classes for large for gestational age neonates. The dashed verticle lines indicate the value of log(λ) corresponding to the first time the whole lipid class was excluded from the model as log(λ) increases. The numbers 1 through 20 indicate the following: (1) covariates; (2) free fatty acids; (3) fatty acid esters of hydroxy fatty acids; (4) cholesterol esters; (5) acylcarnitines; (6) ceramides; (7) glucosylceramides; (8) sphingomyelins; (9) diglycerides; (10) lysophosphatidylcholines; (11) lysophosphatidylethanolamines; (12) phosphatidic acids; (13) phosphatidylcholines; (14) phosphatidylethanolamines; (15) phosphatidylserines; (16) plasmenyl-phosphatidylcholines; (17) plasmenyl-phosphatidylethanolamines; (18) phosphatidylglycerols; (19) phosphatidylinositols; (20) triglycerides.

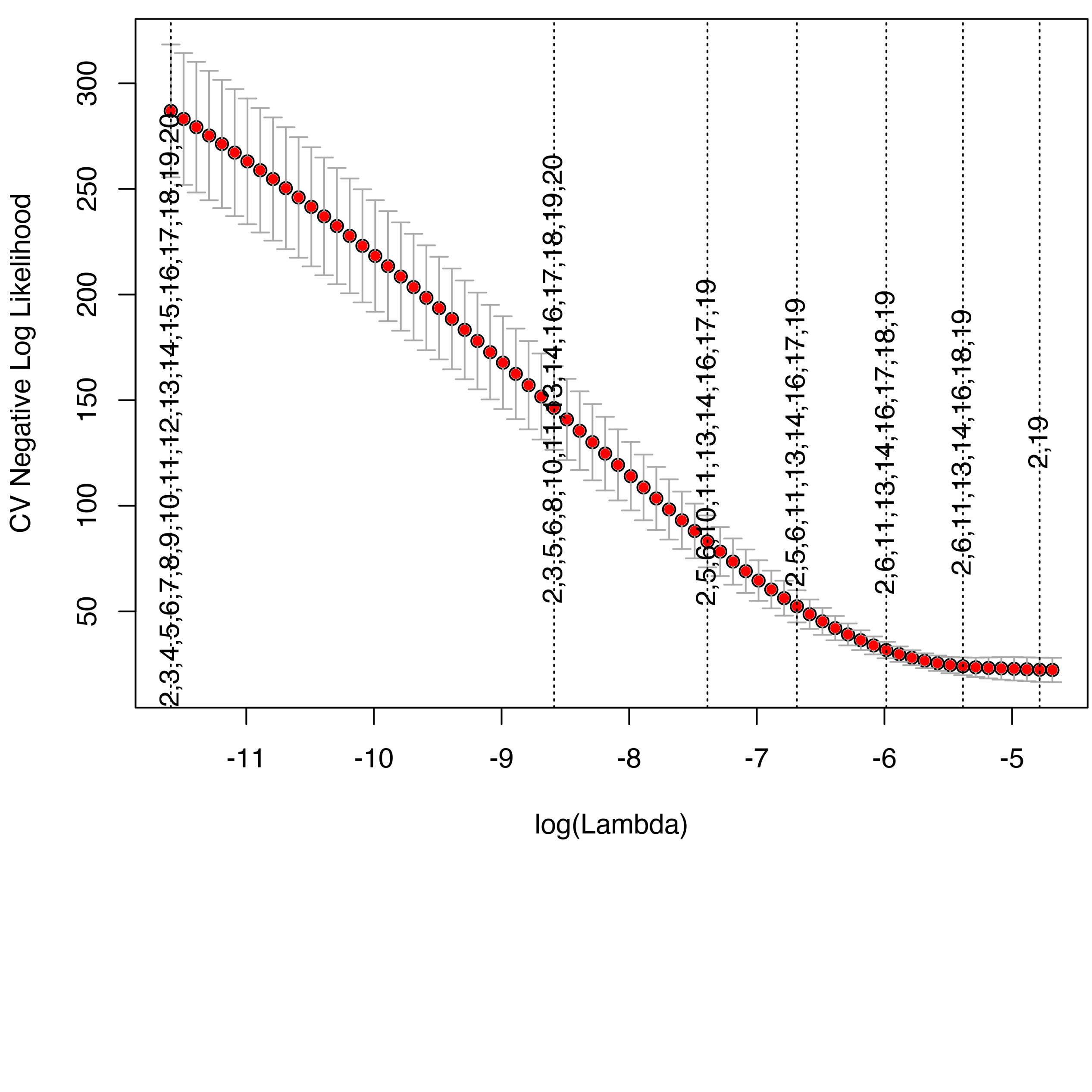

Supplement: Supplementary file 2 — Supplementary Information 2. [file 41598_2021_87472_MOESM2_ESM.pdf]
